# A DNA barcode reference library for endemic Ponto-Caspian amphipods

**DOI:** 10.1038/s41598-022-15442-w

**Published:** 2022-07-05

**Authors:** Denis Copilaş-Ciocianu, Tomasz Rewicz, Arthur F. Sands, Dmitry Palatov, Ivan Marin, Kęstutis Arbačiauskas, Paul D. N. Hebert, Michal Grabowski, Asta Audzijonyte

**Affiliations:** 1 Laboratory of Evolutionary Ecology of Hydrobionts, Nature Research Centre, 08412 Vilnius, Lithuania; 2 Department of Invertebrate Zoology and Hydrobiology, Faculty of Biology and Environmental Protection, University of Lodz, 90-237 Lodz, Poland; 3 Centre for Biodiversity Genomics, University of Guelph, Guelph, ON N1G 2W1 Canada; 4 Area of Ecology and Biodiversity, School of Biological Sciences, University of Hong Kong, Hong Kong, Hong Kong SAR; 5 A.N. Severtsov Institute of Ecology and Evolution of RAS, Moscow, 119071 Russia; 6 Institute for Marine and Antarctic Studies, University of Tasmania, Hobart, Tasmania Australia

**Keywords:** Evolution, Genetics, Zoology

## Abstract

The Ponto-Caspian region is an endemicity hotspot that harbours several crustacean radiations, among which amphipods are the most diverse. These poorly known species are severely threatened in their native range, while at the same time they are invading European inland waters with significant ecological consequences. A proper taxonomic knowledge of this fauna is paramount for its conservation within the native region and monitoring outside of it. Here, we assemble a DNA barcode reference library for nearly 60% of all known Ponto-Caspian amphipod species. We use several methods to define molecular operational taxonomic units (MOTUs), based on two mitochondrial markers (COI and 16S), and assess their congruence with current species-level taxonomy based on morphology. Depending on the method, we find that 54–69% of species had congruent morpho-molecular boundaries. The cases of incongruence resulted from lumping distinct morphospecies into a single MOTU (7–27%), splitting a morphospecies into several MOTUs (4–28%), or both (4–11%). MOTUs defined by distance-based methods without a priori divergence thresholds showed the highest congruence with morphological taxonomy. These results indicate that DNA barcoding is valuable for clarifying the diversity of Ponto-Caspian amphipods, but reveals that extensive work is needed to resolve taxonomic uncertainties. Our study advances the DNA barcode reference library for the European aquatic biota, paving the way towards improved taxonomic knowledge needed to enhance monitoring and conservation efforts.

## Introduction

The advent of DNA barcoding has revolutionized biodiversity research by providing cost-effective and accurate routine species identifications^1^. At the same time, DNA barcoding often reveals the presence of cryptic (morphologically indistinguishable) or pseudo-cryptic (morphologically recognized only after molecular methods have suggested their existence) species within conventionally defined morphospecies^2^. In recent decades, molecular methods, including DNA barcoding, facilitated the discovery of many new taxa by providing relatively objective tools to explore species-level diversity and help address the decreasing level of morphological taxonomic expertise required for biodiversity research^3,4^. The DNA barcoding method is conceptually straightforward since it uses a short DNA fragment of a specific gene (barcode) to identify specimens by comparison with a reference library—the assumption being that these barcodes are species-specific^5^. Due to its ease of use, DNA barcoding has been integrated into various fields ranging from alpha-taxonomy to conservation and invasive species monitoring^6,7^. Nevertheless, its effectiveness depends on publicly available and well curated sequence reference libraries of known and properly identified taxa^8^—one prominent example being the Barcode of Life Database (https://www.boldsystems.org/). However, many taxonomic groups are still underrepresented in these libraries, even in well-studied areas^8,9^. Ideally, species in these libraries should also be represented by multiple sequences originating from geographically distinct populations^10^.

**Figure 1:**
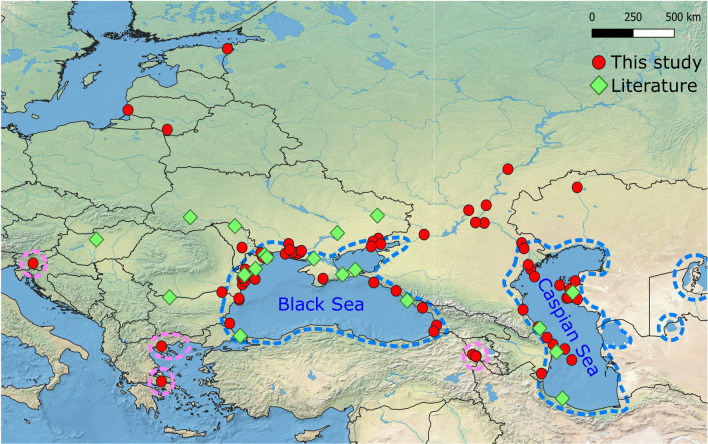
Map of sampling localities. Red dots indicate sampling points from this study while green rhombi indicate literature data. Blue dashed lines indicate the native Ponto-Caspian range. Pink dashed lines indicate areas that contain endemic Ponto-Caspian amphipods which are biogeographically separated from the mother region.

An important geographical area that remains insufficiently studied from a molecular taxonomy and DNA barcoding standpoint is the Ponto-Caspian region, encompassing the Black, Azov, Caspian, and Aral seas, their coastal lagoons, as well as the lower stretches of rivers in their catchments where lower-level salinities currently prevail (Fig. 1). This region harbours a rich endemic fauna known for its tolerance of salinity fluctuations, possibly reflecting adaptations to a turbulent geological history^11,12^. Because of human activities, many Ponto-Caspian species have now dispersed widely across the northern hemisphere, often with significant environmental and ecological consequences^13–15^. Conversely, many Ponto-Caspian endemics are severely threatened within their native range by invasive species, climate change, pollution, and other anthropogenic disturbances^16–21^. The uncertain taxonomic systems for many of these groups hamper accurate biodiversity estimates and appropriate conservation actions^11,22–24^. These pressing issues emphasize the urgent need for a comprehensive barcode reference library for the Ponto-Caspian biota.

Among the endemic Ponto-Caspian evolutionary radiations, crustaceans appear the most diverse with three orders being well represented: Amphipoda, Mysida, and Cumacea^23,25,26^. Amphipods are by far the most speciose, with 96 endemic species currently known, spanning across a broad eco-morphological gradient^23^. Nevertheless, only a fraction of these species have been sequenced^25,27–31^, of these a handful have been studied in a detailed phylogeographic context^32–36^, and even scarcer are modern integrative taxonomic studies^34,37^. Generally, Ponto-Caspian amphipods are poorly studied from evolutionary, ecological, and taxonomic perspectives as most studies focused on a few species of well-known invaders^23^. As a result, Ponto-Caspian amphipod taxonomy is plagued by numerous issues ranging from ambiguous species identity (including invasive ones), to uncertain species diversity at generic or even family levels^23,25,33,34^.

About 40% of the currently recognized Ponto-Caspian amphipod species have now dispersed outside their native range due to shipping, artificial canals interconnecting sea basins and intentional introductions^15,23,38,39^. Their on-going expansion to non-native areas is routinely reported^40–43^, and some taxa have become notorious invaders. For example, the “killer shrimp”, *Dikerogammarus villosus* (Sowinsky, 1894), ranks among the 100 worst invasive species worldwide^44^. These Ponto-Caspian invaders have outcompeted their local counterparts due to their higher aggressiveness, environmental tolerance, increased fecundity, and unpalatability to predators^45–49^. This combination of features has regularly led to the extinction of native species and to the restructuring of ecological communities^50–53^.

This study provides a DNA barcode library for 57 species of Ponto-Caspian amphipods belonging to six families, covering ca. 60% of the taxa known from the region. Using two mitochondrial markers, the standard cytochrome *c* oxidase subunit I (COI) barcoding region and a segment of the large ribosomal RNA subunit (16S rDNA), this study further evaluates the congruence between molecular operational taxonomic units (MOTUs) and the current morphology-based taxonomy. The resulting reference library will help to disentangle the convoluted taxonomic structure of this endemic radiation if integrated with molecular species delimitation methods, morphology, and ecology^23,34,54^. A taxonomically validated reference library will bring three key benefits. First, from a practical standpoint, it will aid the early recognition and monitoring of invasive species that have spread outside their native range, thus enhancing management strategies. Second, it will improve biodiversity estimates for the poorly known endemics and enable their efficient monitoring via non-invasive methods (e.g. eDNA metabarcoding^55^). Lastly, it will provide a basis for future broad-scale, eco-evolutionary and biogeographical studies.

## Results

### Dataset

We obtained 239 COI and 174 16S sequences from 280 specimens and combined them with 31 COI sequences from 8 taxa examined in previous studies (Table 1). The COI alignment had a length of 641 bp, while the 16S alignment was 441 bp including gaps. Among the 57 morphological species in our dataset, 32 were sequenced for COI and 16S fragments for the first time (Table 1). Our dataset contained sequences from all the 15 species that have become broadly distributed outside their native range^39^. Detailed specimen information (i.e. sampling sites, GenBank accession numbers, collectors, etc.) is provided in supplementary Table S1.

**Table 1:** Overview of the 57 species which were studied and their morpho-molecular congruence.

		COI							16S				
Species	Family	BIN	ASAP	PTP	GMYC	KoT = 4	KoT = 5	PDT	ASAP	PTP	GMYC	KoT = 4	KoT = 5
*Akerogammarus* sp.	Gammaridae	OK	OK	OK	OK	OK	OK	OK	OK	OK	OK	OK	OK
Amathillina cristata* Sars, 1894	Gammaridae	OK	OK	OK	OK	S	S	OK	L	L	L	S + L	L
Amathillina pusilla* Sars, 1896	Gammaridae	OK	OK	OK	OK	OK	OK	OK	OK	OK	OK	OK	OK
*Amathillina spinosa* Sars, 1896	Gammaridae	N/A	N/A	N/A	N/A	N/A	N/A	N/A	L	L	L	L	L
*Chaetogammarus hyrcanus* Pjatakova, 1962	Gammaridae	S	OK	S	OK	S	S	OK	OK	OK	S	S	S
Chaetogammarus ischnus* (Stebbing, 1899)	Gammaridae	S	L	L	L	S + L	S + L	L	L	L	L	L	L
*Chaetogammarus placidus* (Sars, 1896)	Gammaridae	OK	OK	OK	OK	OK	OK	OK	OK	OK	OK	OK	OK
*Chaetogammarus sp.*	Gammaridae	OK	OK	OK	OK	S	S	OK	OK	OK	OK	S	OK
*Chaetogammarus warpachowskyi* (Sars, 1894)	Gammaridae	S	S	S	S	S	S	OK	OK	S	S	S	S
*Chelicorophium chelicorne* (Sars, 1895)	Corophiidae	OK	OK	OK	OK	OK	OK	OK	OK	OK	OK	OK	OK
Chelicorophium curvispinum* (Sars, 1895)	Corophiidae	S	L	S + L	L	S + L	L	L	L	L	S + L	S + L	L
*Chelicorophium maeoticum* (Sowinsky, 1898)	Corophiidae	OK	OK	OK	OK	OK	OK	OK	OK	OK	OK	OK	OK
*Chelicorophium monodon* (Sars, 1895)	Corophiidae	L	L	L	L	L	L	L	L	L	L	S + L	L
*Chelicorophium mucronatum* (Sars, 1895)	Corophiidae	S + L	S + L	S + L	S + L	S + L	L	S + L	L	L	L	S + L	L
*Chelicorophium nobile* (Sars, 1895)	Corophiidae	OK	OK	OK	OK	S	S	OK	OK	OK	OK	OK	OK
Chelicorophium robustum* (Sars, 1895)	Corophiidae	S	OK	S	S	S	S	OK	OK	S	S	S	S
*Chelicorophium sowinskyi* (Martynov, 1924)	Corophiidae	OK	OK	OK	OK	OK	OK	OK	OK	OK	OK	OK	OK
Dikerogammarus bispinosus* Martynov, 1925	Gammaridae	S	S	S	S	S	S	S	S	S	S	S	S
Dikerogammarus caspius* (Pallas, 1771)	Gammaridae	OK	OK	OK	OK	OK	OK	OK	OK	OK	OK	OK	OK
Dikerogammarus haemobaphes* (Eichwald, 1841)	Gammaridae	S	S	S	S	S	S	S	L	L	L	L	L
*Dikerogammarus oskari* Birstein, 1945	Gammaridae	N/A	N/A	N/A	N/A	N/A	N/A	N/A	L	L	L	L	L
Dikerogammarus villosus* (Sowinsky, 1894)	Gammaridae	OK	OK	OK	OK	OK	OK	OK	OK	OK	OK	OK	OK
*Echinogammarus karadagiensis* Grintsov, 2009	Gammaridae	OK	OK	OK	OK	OK	OK	OK	OK	OK	OK	OK	OK
*Echinogammarus mazestiensis*†^54^ Marin & Palatov, 2021	Gammaridae	OK	OK	OK	OK	S	OK	OK	N/A	N/A	N/A	N/A	N/A
*Gammaracanthus caspius*†^94^ Sars, 1896	Gammaracanthidae	OK	OK	OK	OK	OK	OK	OK	N/A	N/A	N/A	N/A	N/A
Gmelina aestuarica* Carausu, 1943	Gammaridae	OK	OK	OK	OK	OK	OK	L	N/A	N/A	N/A	N/A	N/A
*Gmelina costata* Sars, 1894	Gammaridae	OK	OK	OK	OK	S	S	L	OK	OK	OK	OK	OK
Gmelinopsis tuberculata* Sars, 1896	Gammaridae	OK	OK	OK	OK	OK	OK	OK	OK	OK	OK	OK	OK
Jugogammarus kusceri* (Karaman, 1931)	Gammaridae	OK	OK	OK	OK	OK	OK	OK	OK	OK	OK	OK	OK
*Lanceogammarus andrussovi* (Sars, 1896)	Gammaridae	OK	OK	OK	OK	OK	OK	OK	OK	OK	OK	OK	OK
*Monoporeia microphtalma* (Sars, 1896)	Pontoporeiidae	OK	OK	OK	OK	OK	OK	OK	OK	OK	OK	OK	OK
*Niphargogammarus aequimanus*†^28^ (Sars, 1895)	Pontogammaridae	L	L	L	L	OK	L	L	N/A	N/A	N/A	N/A	N/A
*Niphargogammarus quadrimanus* (Sars, 1895)	Pontogammaridae	L	L	L	L	OK	L	L	OK	OK	OK	OK	OK
*Obesogammarus boeoticus* (Schellenberg, 1944)	Pontogammaridae	OK	OK	OK	OK	OK	OK	OK	OK	OK	OK	OK	OK
Obesogammarus crassus* (Sars, 1894)	Pontogammaridae	OK	OK	OK	OK	S	S	OK	OK	OK	OK	S	OK
Obesogammarus obesus* (Sars, 1894)	Pontogammaridae	OK	OK	OK	OK	OK	OK	OK	OK	OK	OK	OK	OK
*Obesogammarus platycheir* (Sars, 1896)	Pontogammaridae	OK	OK	OK	OK	OK	OK	OK	OK	OK	OK	OK	OK
*Obesogammarus subnudus* (Sars, 1896)	Pontogammaridae	OK	OK	OK	OK	S	OK	OK	OK	OK	OK	OK	OK
*Onisimus caspius* (Sars, 1896)	Uristidae	S	OK	S	OK	S	S	OK	OK	OK	OK	OK	OK
*Paraniphargoides motasi*†^28^ (Carausu, 1943)	Pontogammaridae	OK	OK	OK	OK	OK	OK	OK	N/A	N/A	N/A	N/A	N/A
Pontogammarus abbreviatus* (Sars, 1894)	Pontogammaridae	L	L	L	L	L	L	L	L	S + L	L	L	L
*Pontogammarus aestuarius* (Derzhavin, 1924)	Pontogammaridae	OK	OK	OK	OK	OK	OK	OK	OK	OK	L	OK	OK
Pontogammarus borceae* Carausu, 1943	Pontogammaridae	S + L	S + L	S + L	S + L	S + L	S + L	S + L	L	S + L	S + L	S + L	S + L
*Pontogammarus cf. aestuarius (Derzhavin, 1924)*	Pontogammaridae	L	L	L	L	L	L	L	L	L	L	L	L
Pontogammarus maeoticus* (Sowinsky, 1894)	Pontogammaridae	S	S	S	S	S	S	S	S	S	S	S	S
Pontogammarus robustoides* (Sars, 1894)	Pontogammaridae	L	L	L	L	L	L	L	L	L	L	L	L
*Pontogammarus sarsi* (Sowinsky, 1898)	Pontogammaridae	OK	OK	OK	OK	OK	OK	OK	OK	OK	OK	OK	OK
*Pontogammarus setosus* (Schäferna, 1914)	Pontogammaridae	OK	L	OK	L	S + L	S + L	L	L	L	OK	L	L
*Shablogammarus shablensis* (Carausu, 1943)	Gammaridae	N/A	OK	OK	OK	OK	OK	OK	OK	OK	OK	OK	OK
Stenogammarus similis* (Sars, 1894)	Pontogammaridae	S	S	S	S	S	S	S	OK	S	S	S	S
*Stenogammarus compressosimilis* Carausu, 1955	Pontogammaridae	OK	OK	OK	OK	OK	OK	OK	OK	OK	OK	OK	OK
*Stenogammarus sp.*	Pontogammaridae	OK	OK	OK	OK	OK	OK	OK	N/A	N/A	N/A	N/A	N/A
Trichogammarus cf. trichiatus (Europe)* (Martynov, 1932)	Gammaridae	OK	OK	OK	OK	OK	OK	OK	OK	OK	OK	OK	OK
*Trichogammarus trichiatus (Caucasus)* (Martynov, 1932)	Gammaridae	S + L	L	L	L	S + L	S + L	L	L	L	L	L	L
Turcogammarus spandli* (Karaman, 1931)	Pontogammaridae	OK	OK	OK	OK	OK	OK	OK	OK	OK	OK	OK	OK
*Wolgagammarus dzjubani* (Mordukhai-Boltovskoi & Lyakhov, 1972)	Pontogammaridae	OK	OK	OK	OK	OK	OK	OK	OK	OK	OK	OK	OK
*Yogmelina cf. limana* (Sars, 1896)	Gammaridae	N/A	N/A	N/A	N/A	N/A	N/A	N/A	OK	OK	OK	OK	OK

Asterisks denote species that have been sequenced in both this study and previously. Daggers indicate species for which sequences were available only from the literature. The remaining species were sequenced for the first time in this study.

*OK* morpho-molecular congruence, *L* lumped, *S* split.

### Species delimitation

**Figure 2:**
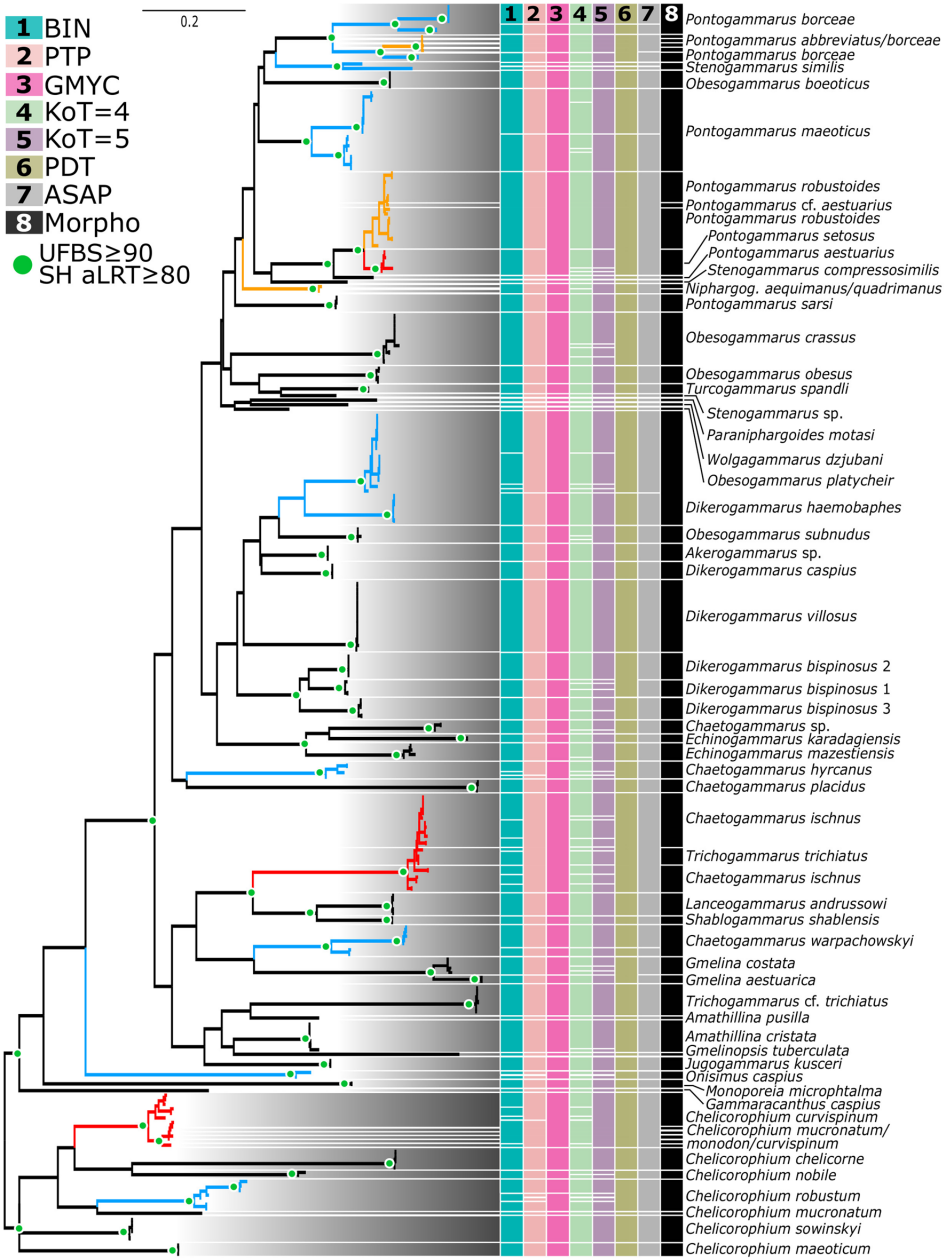
Maximum-likelihood COI tree and species delimitation results. Green dots at nodes indicate strongly supported clades (UFBS – ultrafast bootstrap support; SH aLRT – Shimodaira-Hasegawa approximate likelihood ratio test). The results of the species delimitation analyses are shown by the coloured columns to the right. Each colour represents a different method/parameter setting. Blue branches represent morphospecies which have been split into multiple MOTUs by at least half of the methods. Orange branches indicate morphospecies that have been lumped into one MOTU by at least half of the methods. Red branches indicate morphospecies that were both lumped and split by at least half of the methods. The fully annotated tree is provided in the supplementary Fig. S1.

**Figure 3:**
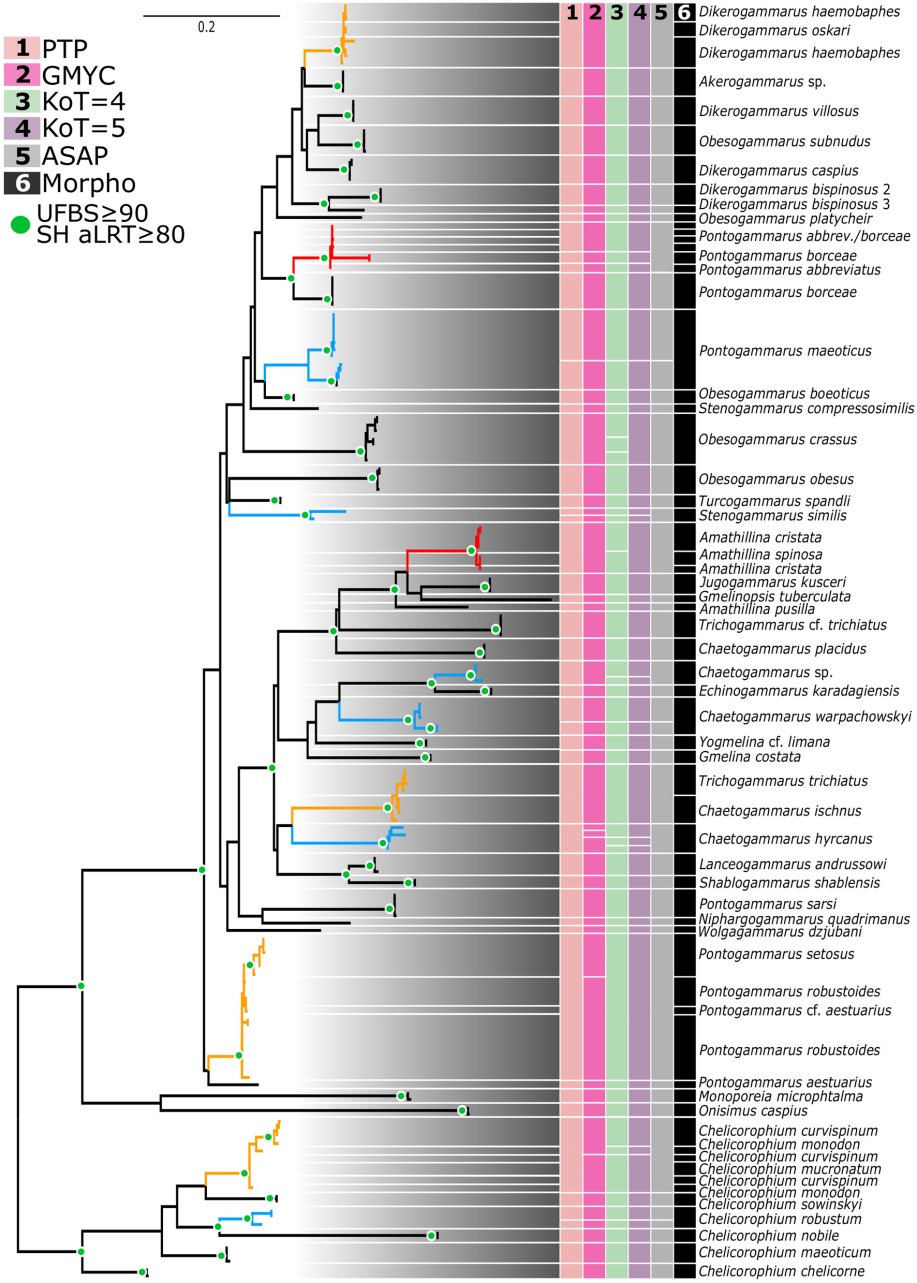
Maximum-likelihood 16S tree and species delimitation results. For details see legend on Fig. 2. The fully annotated tree is provided in the supplementary Fig. S2.

**Figure 4:**
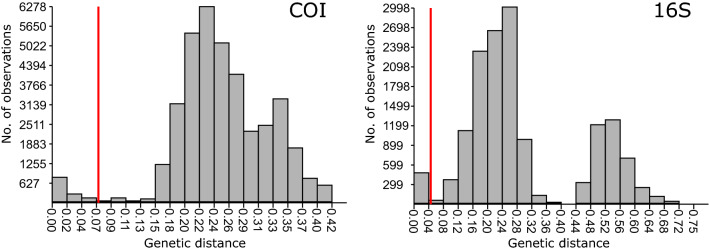
Histogram of COI and 16S K2P distances between specimens. The red line indicates the threshold distance above which specimens are considered to belong to different species, according to ASAP.

We employed six species delimitation methods to define MOTUs and assess their congruence with the current species-level taxonomy based on morphology. The COI and 16S datasets originated from 54 and 51 morphological species (hereafter morphospecies) respectively (Figs. 2, 3, Tables 1, 2). The Assemble Species by Automatic Partitioning (ASAP) method delimited 57 COI and 46 16S MOTUs. The best COI partition had an ASAP score of 2.5 (*p* = 0.00005) at a threshold distance of 0.068 (Fig. 4). The best 16S partition had an ASAP score of 2.5 (*p* = 0.0042) at a threshold distance of 0.055 (Fig. 4). The K/θ (KoT) method with a threshold of 4 (KoT = 4, hereafter) delimited 99 COI and 59 16S MOTUs. With the more conservative threshold of 5, KoT produced 88 COI and 56 16S MOTUs. The Poisson Tree Processes (PTP) method delimited a mean of 64 COI and 51 16S MOTUs, while the Generalized Mixed Yule Coalescent (GMYC) defined 58 COI and 51 16S MOTUs. The patristic distance threshold (PDT) method applied to COI distinguished 54 MOTUs. The Barcode Index Number (BIN) delimitation method revealed 76 COI MOTUs of which 41 are unique. *Shablogammarus shablensis* (Carausu, 1943) did not receive a BIN assignment due to a high number of ambiguous base pairs, although it is divergent enough to be considered a unique BIN. Base pair ambiguity resulted from double peaks in the COI chromatograms. This was not observed for 16S. Given that both studied individuals exhibited the same pattern, it is likely that both COI and a NUMT (nuclear mitochondrial DNA) were recovered.

**Table 2:** Results of congruence between morphology and MOTUs.

Marker/# morphospecies with sequence data	Delimitation method	# MOTUs	% Congruent	% Lump	% Split	% Lump + split
COI/54	BIN	77	66.0	11.3	17.0	5.7
	ASAP	57	68.5	18.5	9.3	3.7
	PTP	64	64.8	14.8	14.8	5.6
	GMYC	58	66.7	18.5	11.1	3.7
	KoT = 4	99	53.7	7.4	27.8	11.1
	KoT = 5	88	53.7	14.8	24.1	7.4
	PDT	54	66.7	22.2	7.4	3.7
	Mean	71	62.9	15.4	15.9	5.8
16S/51	ASAP	46	68.6	27.5	3.9	0.0
	PTP	51	62.7	23.5	9.8	3.9
	GMYC	53	60.8	23.5	11.8	3.9
	KoT = 4	59	56.9	17.6	15.7	9.8
	KoT = 5	56	60.8	25.5	11.8	2.0
	Mean	53	62.0	23.5	10.6	3.9

The comparison between morphospecies and MOTUs revealed congruence ranging from 53.7 to 68.6%, depending on the species delimitation method. The ASAP method showed the highest congruence with morphological taxonomy; its MOTUs matched recognized species in 68.5% of cases for COI and 68.6% for 16S (Table 2). For the KoT = 4 method congruence with morphology was only 53.7% and 56.9% for COI and 16S MOTUs, respectively. The BIN, PTP, GMYC, and PDT methods showed intermediate congruence (Tables 1, 2).

Lumping of distinct morphospecies into one MOTU occurred at frequencies ranging from 7.4% (KoT = 4 at COI) to 27.5% (ASAP at 16S), while the splitting of a morphospecies into several MOTUs occurred at frequencies between 3.9% (ASAP at 16S) to 27.8% (KoT = 4 at COI). There were also a few cases where distinct morphospecies were both split and lumped, but this was uncommon, and only occurring between 0% (ASAP at 16S) to 11.1% (KoT = 4 at COI) of the time (Tables 1, 2).

The overall congruence considering all methods was similar for both molecular markers with a mean congruence of 62.9% for COI and 62% for 16S. Lumping was considerably more common with 16S than COI (23.5% vs. 15.4%), while splitting was more common for COI than 16S (15.9% vs. 10.6%) (Table 2).

The distribution of K2P distances revealed that both markers had a clear barcode gap. As determined with ASAP, specimens diverging at a K2P distance > 7% for COI and > 5–6% for 16S usually belong to different species (Fig. 4). The most common genetic distance between individual pairs was ca. 22–24% at COI and ca. 24–28% at 16S. A bimodal distribution of genetic distances was apparent above the species level, with the second peak mainly corresponding to divergences between species in different families (Fig. 4).

### Misidentification in online databases

When COI sequences obtained in this study were compared with publicly available data from GenBank and BOLD, 14 sequences representing 10 taxa were found to have been misidentified in previous studies (Table 3). Overall, these sequences represent 1.6% of the total publicly available Ponto-Caspian amphipod sequences. In three cases, the misidentified taxa were congeners, but in the remaining cases represented generic misassignments. The most striking case involved a sequence assigned to *Stenogammarus* sp. (MK159944) which did not even cluster with any Ponto-Caspian gammaroid, but belonged to the strictly freshwater *Gammarus balcanicus* Schäferna, 1923 complex according to BLAST searches (99.7% sequence identity). A Neighbor-Joining (NJ) tree with highlighted misidentified sequences is presented in supplementary Fig. S3.

**Table 3:** List of the ten misidentified taxa, their COI GenBank accession number, and their correct identifications.

Misidentified taxon	Accession number	Correct identitfication
*Akerogammarus* sp.	MK159950	*Trichogammarus* cf. *trichiatus*
*Chetogammarus warpachowskyi*	KF478556	*Chaetogammarus ischnus*
*Dikerogammarus haemobaphes*	MK159946	*Pontogammarus robustoides*
*Dikerogammarus villosus*	MK159988	*Dikerogammarus haemobaphes*
*Dikerogammarus villosus*	KF478569	*Dikerogammarus haemobaphes*
*Obesogammarus acuminatus*	KF478574, KT778491, KT778492	*Pontogammarus robustoides*
*Obesogammarus obesus*	AY529039-AY529041	*Pontogammarus abbreviatus/borceae*
*Pandorites podoceroides*	GERA00000000.1	*Obesogammarus crassus*
*Stenogammarus* sp.	MK159944	*Gammarus balcanicus* complex
*Turcogammarus aralensis*	KF478568	*Pontogammarus robustoides*

### Discussion

Our study which is based on a taxonomically diverse dataset and a comprehensive suite of species delimitation methods reveals that ca. 70% of the examined Ponto-Caspian amphipod species show concordance between the MOTUs revealed by DNA barcoding and species recognized through morphological study. However, the remaining species showed morpho-molecular mismatches resulting both from splitting or lumping. Because some of these incongruent taxa are prominent invasive species, our results indicate that taxonomic work is urgently needed to resolve the inconsistencies.

### Taxonomic and evolutionary implications

Many of the taxa (ca. 70%) examined in our study have congruent morphological and molecular boundaries, especially when using the conservative ASAP method (Table 2). This indicates that DNA barcodes can effectively identify Ponto-Caspian amphipods. However, the other species showed consistent morpho-molecular discordances that need further attention. In some instances, the majority of the species delimitation methods indicated the presence of several evolutionary distinct molecular lineages (MOTUs) within a particular morphospecies. Because members of these distinct MOTUs possessed the same diagnostic morphological characters (as defined by Copilaș-Ciocianu and Sidorov^23^), they can provisionally be considered cryptic species^56^. Many cases of cryptic lineages reflected divergences between populations occurring in the Black Sea and Caspian Sea basins, suggesting the importance of geographical isolation for speciation^27^. However, *Pontogammarus borceae* Carausu, 1943 possessed three MOTUs in the Black Sea basin and one in the Caspian Sea. Our study further confirms previous studies that *Dikerogammarus haemobaphes* (Eichwald, 1841), *D. bispinosus* Martynov, 1925, *Pontogammarus maeoticus* (Sowinsky, 1894) and perhaps *Chaetogammarus ischnus* (Stebbing, 1899) contain cryptic lineages that require taxonomic attention^33,34,37^. Interestingly, for amphipods, cryptic or pseudo-cryptic lineages have not yet been detected outside the native range. However, such cases were reported for Ponto-Caspian mysid crustaceans^57^. Despite these cases, it appears that cryptic species are less common within Ponto-Caspian gammarids than in strictly freshwater taxa such as *Gammarus* where most of the nominal species contain numerous cryptic lineages^2,58–60^. Moreover, the morpho-molecular congruence of Ponto-Caspian amphipods is significantly higher than in other lacustrine amphipod radiations such as in Lake Ohrid or Titicaca, where extensive mismatches were observed^61,62^.

Apart from these splits, we also observed cases where several distinct morphospecies were merged into a single MOTU. The most remarkable case involved *Trichogammarus* (alternatively *Echinogammarus*) *trichiatus* (Martynov, 1932) which was nested within *C. ischnus*. Earlier studies sequenced European populations of *T. trichiatus,* but no prior sequences came from the Caucasian range, which is the type locality of *T. trichiatus*^28,63^. Our results show that the European taxon (indicated as *T. cf. trichiatus*) is molecularly distinct, and likely represents a distinct species from the Caucasian taxon (indicated as *T. trichiatus*). The European taxon was also initially described as a separate species (from Romanian and Bulgarian lagoons), but was later synonymized with the Caucasian *T. trichiatus*^63^. Other cases of lumping involved *Pontogammarus robustoides* (Sars, 1894) and *P. setosus* (Schäferna, 1914), *Amathillina cristata* Sars, 1894 and *A. spinosa* Sars, 1896*,* and *D. haemobaphes* and *D. oskari* Birstein, 1945 (Figs. 2, 3). The first taxa pair is allopatric while the last two pairs are sympatric. In one exceptional instance, DNA markers showed that the three currently described morphospecies *Chelicorophium curvispinum* (Sars, 1895), *C. monodon* (Sars, 1895), and *C. mucronatum* (Sars, 1895) (Figs. 2, 3) are likely to belong to one species. None of these cases appear to represent mitochondrial introgression as the same lack of divergence was observed for the nuclear markers (unpublished data), indicating either incipient speciation or that some “species” are just ecophenotypic variation.

In rare cases, we observed both lumping and splitting of a particular morphospecies. For example, *D. haemobaphes* was split into two cryptic lineages but *D. oskari* was nested in one of them. Similarly, *P. borceae* was split into three lineages in the Black Sea, but its Caspian lineage was lumped with *P. abbreviatus* (Sars, 1894). *Chelicorophium mucronatum* was sometimes resolved as a distinct lineage, but in other cases it was lumped with *C. curvispinum* and *C. monodon*.

These inconsistencies between MOTUs and morphology indicate the need for integrative taxonomic work, preferably incorporating nuclear and mitochondrial markers, as well as morphology and ecology^64^. Our study does affirm the value of DNA barcoding as an important first step for exposing cases of taxonomic incongruence. From an evolutionary perspective, the high and low genetic distances observed between and within morphospecies might indicate that the Ponto-Caspian amphipod fauna has been diversifying in complex ways and at differing rates.

### Reference library and importance of accurate species identifications

Although our study has more than doubled the DNA barcode coverage for Ponto-Caspian amphipods by adding records for 32 taxa, the current dataset only includes representatives for ca. 60% of the known fauna. Nevertheless, the current data includes the most common species and all species that have substantially dispersed outside their native Ponto-Caspian realm. Therefore, the data from this study constitutes a reliable reference library for monitoring and early detection of species that have invaded Europe and North America. It also provides a foundation for further taxonomic progress.

By comparing our COI dataset with sequences available in GenBank and BOLD, we detected 14 cases of misidentification or errors in data entry. In some cases, the incorrectly identified species are morphologically similar to the correct identification (e.g. *D. haemobaphes* misidentified as *D. villosus*). However, most cases of misidentification involve representatives of different genera that are very distinct morphologically, e.g. *T.* cf. *trichiatus* misidentified as *Akerogammarus* sp., or *Obesogammarus crassus* (Sars, 1894) misidentified as *Pandorites podoceroides* Sars, 1895 (Table 3, Fig. S1). Such cases may reflect errors in data handling or contamination. Sequencing of immature specimens might also be the source of some errors since their accurate identification is more problematic. However, these errors clearly emphasize the need to reverse the trend in declining numbers of well-trained taxonomists^65^.

Because well-validated DNA barcode reference libraries are critical for accurate taxonomic species identification via barcoding or metabarcoding, the occurrence of misidentified/mislabeled sequences is highly problematic, especially for taxa with few sequences available. For example, prior to our study there was only one publicly available COI sequence labeled as *Chaetogammarus warpachowskyi* (Sars, 1894), however, we show that this is in fact a misidentified specimen of *C. ischnus*. There needs to be an on-going effort to ensure that records in reference libraries are carefully validated.

### Accuracy of species delimitation methods

Our results indicated that MOTUs delineated by the conservative ASAP method, which clusters individuals into species based on the gap between intra- and interspecific genetic distances, had the highest congruence with morphology-based taxonomy, with values approaching 69% for both the COI and 16S markers (Table 2). However, on the downside, it lumped more morphospecies than the other methods, especially for the more conserved 16S marker (28%). The COI-based patristic distance threshold (PDT) and BINs showed similar congruence levels at 67% and 66%, the former being more prone to lumping and the latter to over splitting. The congruence levels of the tree-based PTP and GMYC methods were also not far off, with 65% and 67% for COI, and 63% and 61% for 16S. For PTP, lumping and splitting occurred with similar frequency at COI but lumping was twice as prevalent at 16S. For GMYC, lumping was almost twice more prevalent than splitting for both markers. However, the KoT method at the traditional level of 4 was obviously much less congruent with morphology (54% for COI and 57% for 16S). With respect to markers, it was more prone to over splitting at COI and to lumping at 16S. Using a more conservative threshold of 5 increased the accuracy, but only for 16S.

These present results are in broad agreement with previous studies, as they showed that distance-based methods, without a priori divergence thresholds, were generally more accurate than tree-based approaches which can be more prone to over splitting^66–69^. The patristic distance threshold of 0.16 substitutions per site proposed for the COI marker^70^ is rather realistic and should be employed more often in crustacean taxonomy, although it may be overly conservative in some instances^71^.

## Conclusions

In summary, this study provides COI barcodes and 16S rDNA sequences for 57 morphospecies of Ponto-Caspian amphipods, including all the species that have colonized habitats outside their native geographical range. The results confirm that DNA barcoding is an effective tool for identifying Ponto-Caspian amphipod species, with distance-based species delimitation methods without a priori divergence thresholds showing the strongest congruence with morphology-based taxonomy. This study also revealed misidentified records in public sequence reference libraries and the presence of cryptic diversity and taxonomic inconsistencies in some species that require further attention. As a result, this study makes an important contribution to the European DNA barcode reference library for aquatic biota, thus paving the way towards improved taxonomic knowledge needed to support biomonitoring and conservation efforts.

## Methods

### Sampling and data collection

As Ponto-Caspian amphipods occur over a broad depth range^23^, specimens were collected by a range of methods, aimed to target different habitats and depths. Shoreline species were collected by hand-picking or kick sampling with a hand net. Species occurring in deeper offshore-waters were collected off boats by either dredge or grab sampler. All material was preserved in 70–96% ethanol. Geographically, sampling efforts spanned across most of the Ponto-Caspian region, but also included areas outside this mother range where some of the species are either native (e.g. Balkan Peninsula and the Caucasus) or non-native (e.g. the Baltic basin). We generally aimed to represent each species by several sequences (mean = 5, range 1–24) from various geographical localities, preferably from both the Black Sea and Caspian Sea basins, as different basins are known to harbour distinct lineages^25,34,72^. Amphipods were identified using the appropriate keys^23,73–76^.

Samples were processed for sequencing either at the Nature Research Centre, Vilnius, Lithuania, at the Canadian Center for DNA Barcoding (CCDB), Guelph, Canada, or at the Department of the Invertebrate Zoology and Hydrobiology, University of Lodz, Lodz, Poland (Table S1).

Samples analysed at the Nature Research Centre were processed as follows. DNA was obtained either from entire animals (if smaller than 6 mm) or from tissue from the dorsal portion of the body excised using a biopsy punch. DNA was isolated using the Quick-DNA Miniprep Plus Kit (Zymo Research) following manufacturer’s instructions with the lysis step prolonged overnight. Afterwards, the cuticle of entire specimens was placed in 95 °C distilled water for 5 min to inactivate the enzymes. Subsequently it was stained with azophloxin (Sigma-Aldrich) in 70% ethanol overnight at 80 °C. Lastly, it was placed in a 3:1 mixture of 70% ethanol and glycerol for long-term preservation as a specimen voucher.

Two mitochondrial fragments were targeted for PCR amplification. First, we amplified a 641 bp fragment (Folmer region) of the cytochrome *c* oxidase subunit one (COI) using the universal primers LCO1490-JJ and HCO2198-JJ^77^ with the following thermal cycling protocol: 1 cycle of initial denaturation at 95 °C for 60 s; 39 cycles of denaturation at 95 °C for 30 s, annealing at 49 °C for 60 s, and extension at 72 °C for 60 s; 1 final extension cycle of 72 °C for 5 min. Second, we amplified a ca. 380 bp region of the large ribosomal RNA subunit (16S) using the primers 16STf^30^ and 16Sbr^78^ following the PCR protocol in Copilaș-Ciocianu and Petrusek^79^. PCR reactions were performed in 25 μL volumes, consisting of 1 μL DNA template, 1 μL of each primer, 12.4 μL of DreamTaq PCR Master Mix (Thermo Scientific) and completed with 10 μL of nuclease-free water. Reaction products were visualized on a 2% agarose gel to confirm amplification. Where the template had a low DNA concentration and gel bands were weak, a second nested PCR was performed with 2 μL of the first PCR serving as a template. Positive amplicons were unidirectionally sequenced commercially by BaseClear using the PCR primers. In cases where the chromatograms were short or of poor quality, bidirectional sequencing was performed.

For samples analysed in the CCDB and University of Lodz, the detailed methodology regarding DNA extraction, amplification, and sequencing was provided in Morhun et al.^34^ and Csabai et al.^40^ The obtained sequences are available in GenBank (COI accession numbers: ON257912– ON258156; 16S accession numbers: ON258157–ON258328, ON668379, ON668380) and the Barcode of Life Database (http://dx.doi.org/10.5883/DS-DCCPC) (Table S1). The new COI records were supplemented with 31 sequences from 8 taxa obtained from GenBank to increase taxon coverage (see Supplementary Table S1). Sequence alignments are available on Figshare (https://doi.org/10.6084/m9.figshare.20071748).

### Sequence alignment and phylogenetic trees

Cytochrome *c* oxidase subunit one sequences were aligned with MUSCLE^80^ in MEGA 6^81^, while the 16S sequences were aligned with MAFFT 7^82^ using the G-INS-i iterative refinement method. The protein-coding COI alignment was amino-acid translated to check for premature stop codons that would indicate pseudogenes (none were observed). All sequences were screened for contamination using BLAST (https://blast.ncbi.nlm.nih.gov/Blast.cgi).

For each marker we constructed a maximum-likelihood (ML) phylogeny with W-IQ-TREE^83^ (http://iqtree.cibiv.univie.ac.at/), using the GTR + I + Γ model (determined with MEGA 6) and 1000 ultrafast bootstrap replicates. This analysis sought only to provide a glimpse into phylogenetic relationships among taxa, not to critically assess phylogenetic patterns which would have required much more extensive sequence analysis.

### Species delimitation analyses

To assess the congruence between lineages identified as distinct through morphology and molecular analysis, we used a suite of species delimitation methods to ascertain molecular operational taxonomic units (MOTUs). These methods included both distance- and tree-based species delimitation methods with or without an a priori divergence threshold. The distance-based methods included three approaches: (1) a non-threshold method that uses a hierarchical clustering algorithm to identify a barcode gap (i.e. difference between intra and interspecific genetic distances)—ASAP (Assemble Species by Automatic Partitioning^67^), (2) a threshold method that calculates the ratio between the average interspecific distance (*K*) of two clades and their intraspecific genetic diversity (θ)—KoT (K/θ^84^), and (3) Barcode Index Number (BIN), which is an algorithm implemented in Barcode of Life Data Systems (BOLD; https://www.boldsystems.org/), where the newly submitted COI sequences are aligned and compared pairwise and also to each sequence already deposited in BOLD. The tree-based methods included three approaches: (1) a non-threshold method that distinguishes coalescent versus speciation processes on a phylogram assuming a Poisson distribution—PTP (Poisson Tree Processes^85^), (2) a non-threshold method that determines the transition between species-level to population-level evolutionary processes by using a generalized Yule model—GMYC (Generalized Mixed Yule Coalescent^86^), and (3) a proposed molecular threshold for crustaceans which assumes that clades separated by a patristic distance of at least 0.16 substitutions per site at the COI locus are likely different species—PDT (Patristic Distance Threshold^70^). All these methods were applied to both markers, except for PDT and BIN which were applied only to COI as they were developed specifically for this marker^70^.

For the ASAP method (implemented on https://bioinfo.mnhn.fr/abi/public/asap/), we used default settings and Kimura 2-parameter (K2p) distances. For the KoT method (implemented on https://eeg-ebe.github.io/KoT/), we used two distance thresholds. First we used a threshold of four, meaning that interspecific divergence is at least four times greater than intraspecific divergence (the 4 × rule^87^), and, to be more conservative, we repeated the analyses with a threshold of five. For the PTP method (implemented on https://species.h-its.org/), we used the ML gene trees generated with IQTREE (see previous section). These analyses were run for 500,000 generations, a thinning of 100, and a 0.1 burn-in. For the PDT method we used the same ML tree along which patristic distances were calculated using the Patristic 1.0 software^88^. We used the GMYC model as implemented in iTaxoTools 0.1^89^. As input we used ultrametric trees computed with BEAST 1.8.2^90^ using the GTR + I + Γ model for each marker, a Birth–Death speciation prior, and a strict clock rate. The analyses were run for 50 million generations and the first 10% of the trees were discarded as burn-in. Convergence statistics and effective sample sizes were assessed with Tracer 1.6^91^.

### Detection of misidentified sequences in GenBank

While comparing our data with previously published sequences, we detected several inconsistencies regarding the taxonomic placement of public sequences. As a result, we conducted a more detailed investigation to clarify the potential misidentifications or mislabelling. We searched GenBank (https://www.ncbi.nlm.nih.gov/genbank/) and BOLD (https://www.boldsystems.org/) for all available COI sequences following our previously published species checklist^23^. We downloaded 840 sequences that could be aligned with our dataset. The COI sequence of “*Pandorites podoceroides*” was obtained by Macher et al.^92^, and originated from the transcriptome data obtained by Naumenko et al.^93^. The final dataset containing both our original and published data totalled 1079 sequences. The sequences were aligned in MAFFT 7^82^ (https://mafft.cbrc.jp/alignment/server/) with default settings. Amino-acid translation confirmed the global homology of the alignment and revealed no premature stop codons. A Neighbour-Joining (NJ) tree was constructed in MEGA 6 based on the number of differences, using uniform rates and 100 bootstrap replications. Subsequently, this tree was manually inspected for mislabelled/misidentified sequences by checking for anomalous clustering patterns (i.e. sequences of species X clustering within species Y).

## Supplementary Information

Supplementary Information 1.

Supplementary Information 2.

Supplementary Information 3.

Supplementary Information 4.

## Data Availability

The datasets generated and/or analysed during the current study are available in the BOLD repository (http://dx.doi.org/10.5883/DS-DCCPC) and in GenBank (COI accession numbers: ON257912–ON258156; 16S accession numbers: ON258157–ON258328, ON668379, ON668380). Sequence alignments are available on Figshare (https://doi.org/10.6084/m9.figshare.20071748).
