# Supplementary figures and images for: A DNA barcode reference library for endemic Ponto-Caspian amphipods
Source: Sci Rep. 2022 Jul 5;12:11332. doi: 10.1038/s41598-022-15442-w (PMC9256591; doi:10.1038/s41598-022-15442-w)

- 1** BIN  
**2** PTP  
**3** GMYC  
**4** KoT=4  
**5** KoT=5  
**6** PDT  
**7** ASAP  
**8** Morpho  
 ● UFBS $\geq 90$   
 ● SH aLRT $\geq 80$

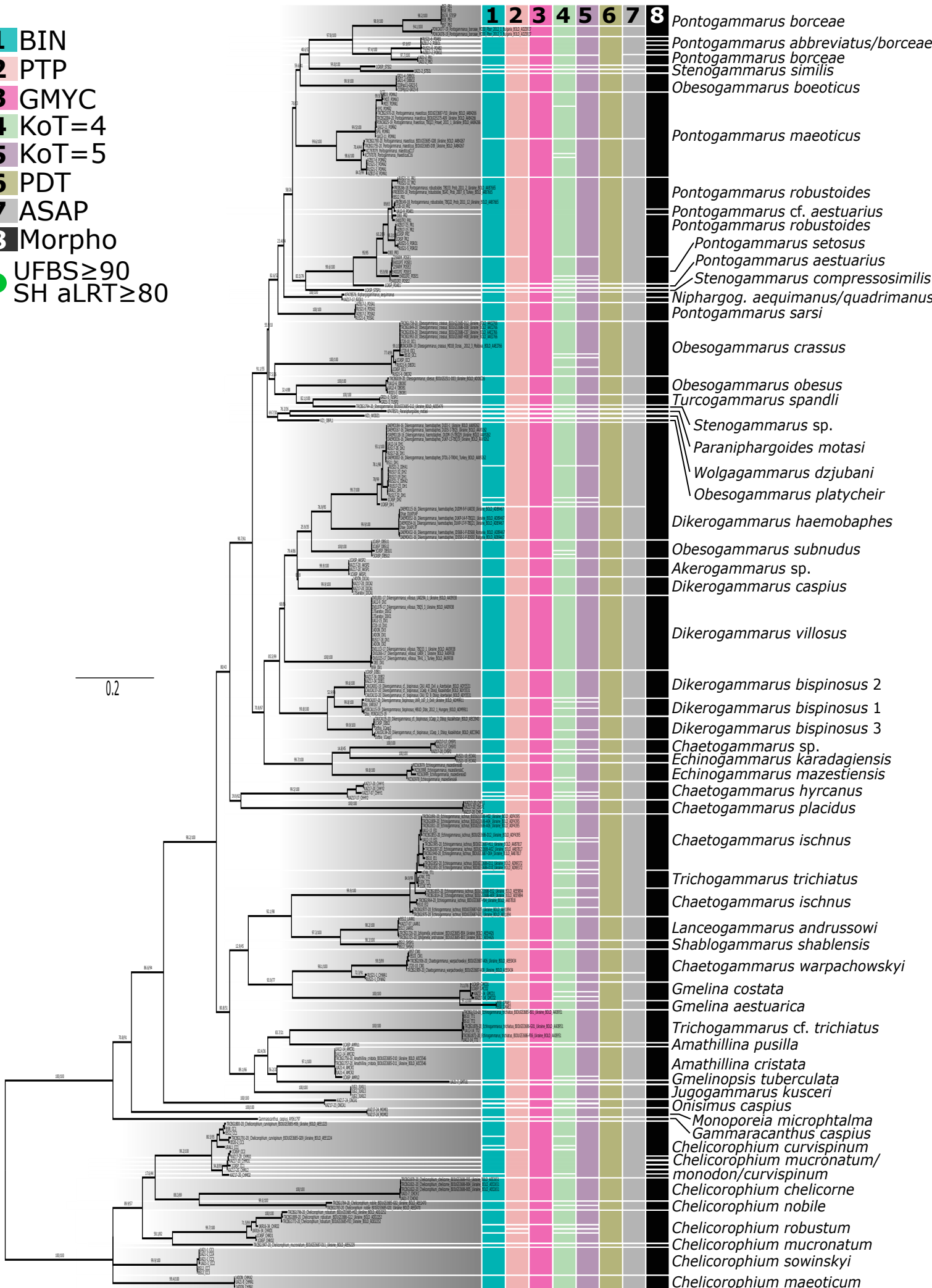

Supplement: Supplementary file 1 — Supplementary Information 1. [file 41598_2022_15442_MOESM1_ESM.pdf]

- 1** PTP  
**2** GMYC  
**3** KoT=4  
**4** KoT=5  
**5** ASAP  
**6** Morpho

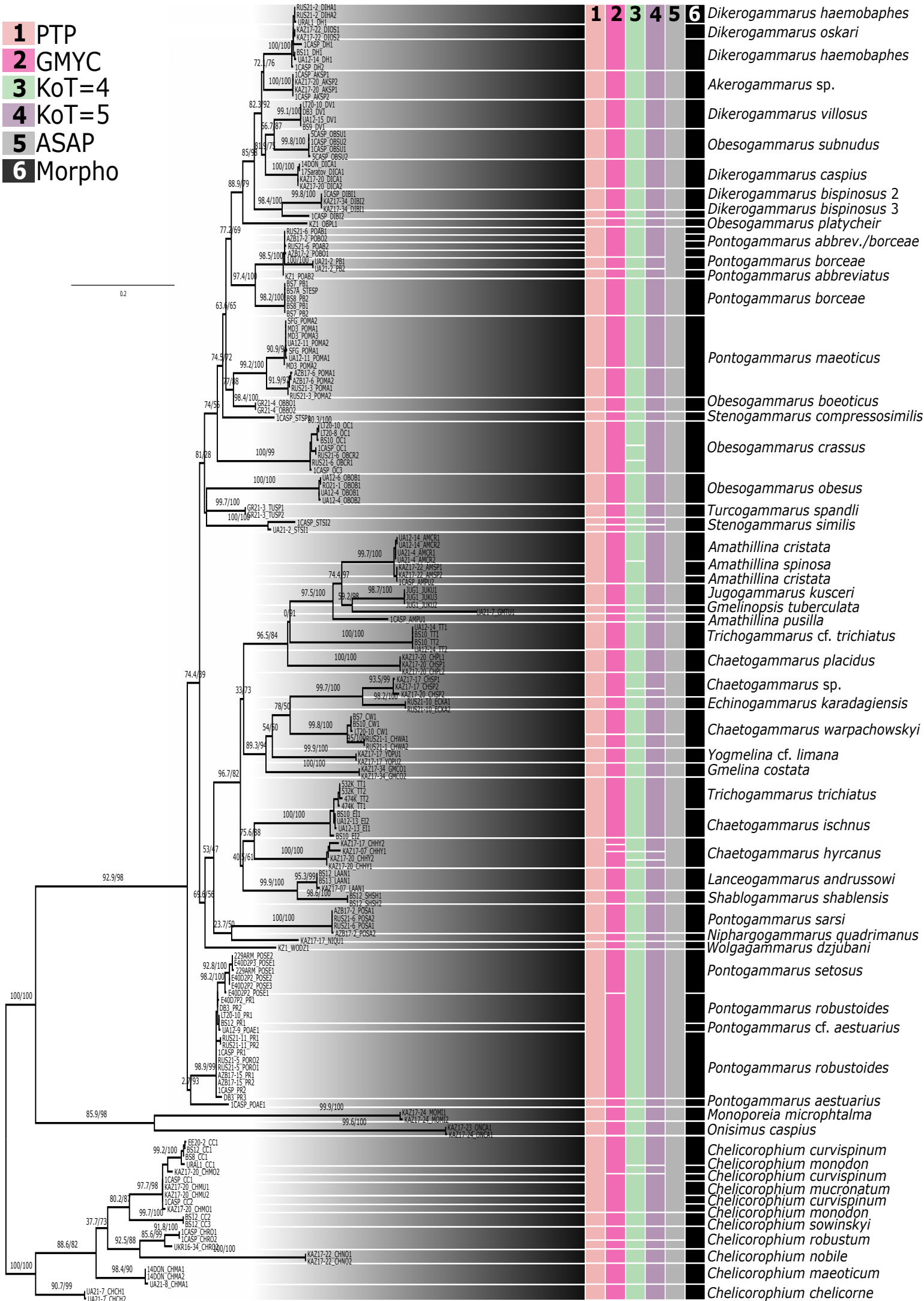

Supplement: Supplementary file 2 — Supplementary Information 2. [file 41598_2022_15442_MOESM2_ESM.pdf]

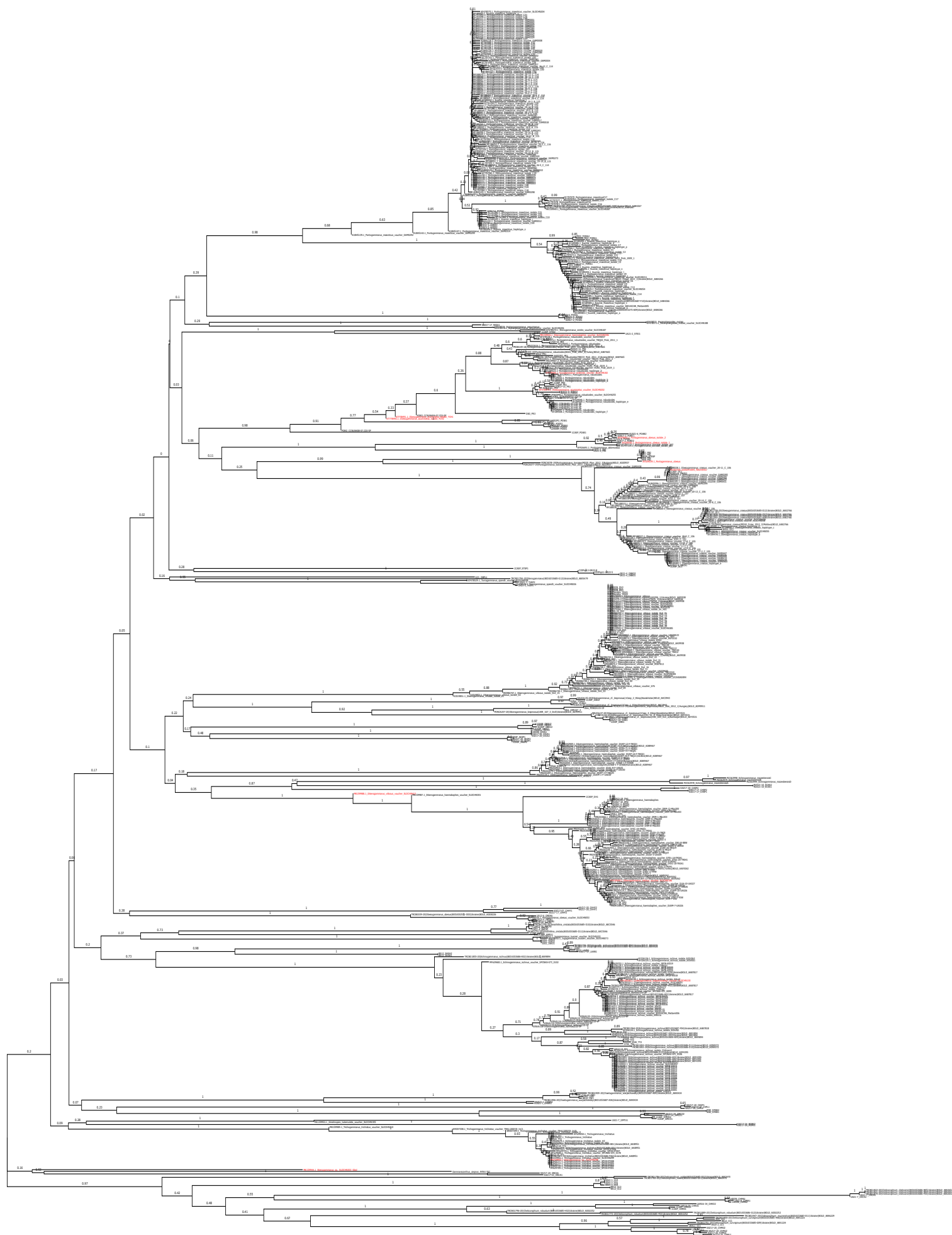

Supplement: Supplementary file 3 — Supplementary Information 3. [file 41598_2022_15442_MOESM3_ESM.pdf]
